# A prospective observational study exploring the association of comorbid chronic health conditions with total healthcare expenditure in people with mental health conditions in an Asian setting

**DOI:** 10.1186/s12888-022-03827-0

**Published:** 2022-03-19

**Authors:** Shilpa Tyagi, Ganga Ganesan, Mythily Subramaniam, Edimansyah Abdin, Janhavi Ajit Vaingankar, Boon Yiang Chua, Siow Ann Chong, Kelvin Bryan Tan

**Affiliations:** 1 MOH Office for Healthcare Transformation, Harbourfront Centre, Maritime Square, Singapore, 099253 Singapore; 2 Policy, Research and Evaluation Division, Ministry of Health, Singapore, Singapore; 3 Research Division, Institute of Mental Health, Singapore, Singapore

**Keywords:** Mental health conditions, Healthcare expenditure, Comorbidity

## Abstract

**Background:**

It is pertinent to focus on chronic medical condition (CMC) comorbidity with mental health conditions (MHC) as their co-occurrence has significant cost and health implications. However, current evidence on co-occurrence of MHC with CMC is mixed and mostly from Western settings. Therefore, our study aimed to (i) describe the association between MHC and total healthcare expenditure, (ii) examine the association between CMC and total healthcare expenditure and (iii) examine determinants of total and different types of healthcare expenditure in respondents with and without MHC in an Asian setting.

**Methods:**

The data from Singapore Mental Health Study (SMHS) 2016, a nationwide epidemiological survey, were linked with the National claims record (from 2017 to 2019). Multivariable Generalized Linear Models (GLM) were used to examine the association between MHC and total and different types of healthcare expenditure.

**Results:**

A total of 3077 survey respondents were included in current analysis. Respondents with MHC had a lower mean age of 38.6 years as compared to those without MHC (47.1 years). MHC was associated with increased total healthcare expenditure after adjusting for covariates (b = 0.508, *p* < 0.05). In respondents with MHC, presence of CMC increased the total healthcare expenditure by 35% as compared to 40% increase in those without MHC. Interestingly, 35–49 years age group with MHC had almost 3 times higher total healthcare expenditure and 7.5 times higher inpatient expenditure as compared to the 18–34 years age group.

**Conclusion:**

Our study highlights variations in association of CMC and age with total healthcare expenditure in those with versus without MHC in an Asian setting. Practical recommendations include appropriate planning and resource allocation for early diagnosis and management of MHC, proactive screening for CMC in those with MHC and addressing the dual issues of treatment gap and stigma to facilitate early help seeking and prevent episodic, costly healthcare utilization.

**Supplementary Information:**

The online version contains supplementary material available at 10.1186/s12888-022-03827-0.

## Background

Globally, mental health conditions (MHC) pose a significant burden to the healthcare system, with mental and behavioural disorders accounting for 7.4% of disability-adjusted life years (DALYs) and 22.7% of years lived with disability (YLD) [1]. Within Singapore, according to a nationwide survey conducted in 2010, about 12% of the population had at least one lifetime mood, alcohol use or anxiety disorder [2]. MHCs are often co-occurring with comorbid conditions, with mental and physical comorbidity associated with poorer quality of life and higher work-lost days as compared to those without any mental and physical disorders [3]. Studies in Canada, England and Germany have also shown significantly higher healthcare resource utilization and associated expenditure in respondents with MHC as compared to those without MHC [4–6]. To our best knowledge, there are little or no studies of this phenomenon in Asian countries.

One of the key concerns is whether MHCs are associated with increased healthcare expenditure and whether this is attributable solely to the management of MHCs, or whether it is due to increased healthcare expenditure on other comorbid conditions. The co-occurrence of MHC with chronic medical condition (CMC) could be additive, resulting in increased healthcare expenditures, or synergistic, resulting in lower expenditures. The evidence in the literature is currently not well established. For instance, a US-based study reported being treated for depression is associated with reduced overall costs and diabetes attributable costs in patients with co-existing diabetes, depression and other chronic conditions [7]. On the contrary, another study reported co-occurrence of depression and individual chronic conditions was associated with increased total healthcare expenditures [8]. Testing the additional cost effect of comorbid health conditions on total healthcare expenditure, a previous study showed that the cost effect was smaller rather than larger for individuals with severe mental illness [9]. This study was unable to ascertain whether this is due to comparatively limited access to healthcare for these individuals or better coordination between medical and mental health providers which may reduce cost.

None of these studies were from an Asian setting in which help seeking behaviour of people with MHC may be systematically different from Western settings due to social and cultural differences. To address this knowledge gap, we use the Singapore Mental Health Study (SMHS) conducted in 2016, linked with 3 years of administrative data on acute hospital and outpatient utilisation from 2017 to 2019 to examine the healthcare expenditure burden of MHC. Our study aims to (i) describe the association between the presence of MHC and total healthcare expenditure, (ii) examine the association between CMC and total healthcare expenditure in respondents with and without MHC and (iii) examine the differences in determinants of total and different types of healthcare expenditure in respondents with and without MHC in an Asian setting.

## Methods

### Data source and sample

The SMHS was a nationwide epidemiological survey conducted in 2016 to describe the mental health of the population. All Singaporeans and permanent residents aged 18 years and above were eligible to participate in the SMHS. The detailed methodology of the survey is described elsewhere [10]. Participants were approached in their homes, and written informed consent was taken before commencing the data collection. The interviews were conducted in different languages based on participants’ preferred language (i.e., English, Chinese or Malay). Trained interviewers administered the study survey using a tablet, with such interviews lasting approximately for 1.5 to 2 h. The SMHS was approved by the institutional review board (National Healthcare Group Domain Specific Review Board, Reference Number: 2015/01035 NHG DSRB).

Participants were explicitly asked as part of the written informed consent if they were willing to link the survey data to data in administrative databases maintained by the Ministry of Health (MOH), Singapore. 3085 participants agreed to the data linkage, and for this sub-sample, the SMHS 2016 data were linked with 3 years of administrative data (from 2017 to 2019) obtained from the National claims record to study the healthcare expenditure burden of the mental health conditions. National claims record is a “nation-wide database of healthcare services utilization and associated costs” under the MOH, Singapore [11]. This linkage was achieved via a unique identification number assigned to all Singaporean citizens and permanent residents and is widely used in all administrative contexts, including healthcare utilization. We achieved a match rate of about 99% across both databases using this unique identification number for the current study. Henceforward, we will use ‘administrative data’ to denote the National claims record and ‘survey data’ to denote the SMHS 2016 data. While most of the independent variables were taken from the survey data, the dependent variables were taken from the administrative data.

### Study variables

Mental health disorders were diagnosed using the fully structured, computer-assisted version of the Composite International Diagnostic Interview version 3.0 (WHO CIDI 3.0) [12]. The participants were assessed for the following mental health conditions: mood disorders (major depressive disorder or MDD, dysthymia, bipolar disorder), anxiety disorders (generalised anxiety disorder (GAD) and obsessive compulsive disorder (OCD)) and alcohol use disorders (alcohol abuse and alcohol dependence). Among participants of survey data (for whom presence of MHC were determined) the CMCs or physical conditions were determined using both survey data and administrative data as researchers have previously recommended relying on multiple data sources to determine the presence of chronic health conditions [13]. For the current analysis, the CMC included arthritis, asthma, cancer, chronic obstructive lung disease, diabetes, epilepsy, heart disease, heart failure, hyperlipidaemia, hypertension, Parkinson’s disease, peptic ulcer disease, renal failure, stroke and thyroid disease.

The dependent variables were total healthcare expenditure (inclusive of subsidies, insurers’ payments and out-of-pocket payments) per year from 2017 to 2019, including both the acute and outpatient services and healthcare expenditure related to specific services (i.e., acute services including inpatient and emergency department (ED) costs and outpatient services including primary care (PC) and specialist outpatient care (SOC) costs). Costs related to inpatient hospitalizations (excluding maternal and perinatal admissions) and ED visits were included under acute services. Costs related to PC services and SOC services (excluding obstetrics specialty) were included under the outpatient services. PC comprised of both public and private PC services. While public PC included all government run polyclinics or “one-stop primary care clinics” which offer a comprehensive range of services including clinical, health education, diagnostic and pharmacy services [14], private PC only included those general practitioner (GP) clinics which were enrolled in the Community Health Assist Scheme (CHAS) Programme. CHAS is a national subsidy scheme by Ministry of Health (MOH) which provides low and middle-income Singaporeans and permanent residents portable subsidies to pay for GP services [15].

### Statistical analysis

Descriptive statistics were calculated to describe the sample characteristics using proportion or mean (SD) for categorical and continuous variables, respectively. Multivariable Generalized Linear Models (GLM) were used to examine the association between MHC and total healthcare expenditure. Gamma distribution with a log link function was used for our outcome variable of cost. The model specifications were informed by prior literature on healthcare expenditures [16–18]. The selection of covariates was guided by clinical relevance, information availability and prior literature [4, 5, 19]. Model 1 or unadjusted base model included only MHC variable as the independent variable and total healthcare expenditure as the dependent variable. Subsequently, Model 2 and Model 3 were implemented as adjusted models incorporating age only and all covariates (age, sex, race, education and number of CMC), respectively, in the base model or Model 1. We added an interaction term between MHC and the number of CMC in Model 3 to examine the differences in the association of CMC with total healthcare expenditure in the presence of MHC compared to in the absence of MHC. To get a deeper understanding of the predictors of total healthcare expenditure in those with MHC, we ran separate adjusted models for respondents with MHC (Model 4) and without MHC (Model 5), respectively.

We also examined the predictors of specific healthcare services (i.e., acute services including inpatient and ED costs and outpatient services including PC and SOC costs) related expenditures in those with MHC and without MHC. The significance level was set at 5%. The alpha level was set at 0.05 for the analyses. All statistical analysis was performed in Stata 16 [20].

## Results

There were 6126 respondents in the SMHS 2016, out of which 3085 consented to the linkage of their survey data to administrative data. A total of 3077 survey respondents with the successful matching of both databases were included in the current analysis, with 511 (16.6%) with a MHC and 2566 (83.4%) without any MHC. While among respondents without MHC, 1508 (59%) had a CMC, among respondents with MHC, 278 (54%) had a CMC.

Table 1 shows the sample characteristics stratified by those without and with MHC. The group of respondents with MHC had a lower mean age of 38.6 years as compared to those without MHC (47.1 years). While 46% of the respondents with MHC were 18 to 34 years old, this proportion was only 28% in the group without MHC. The group with MHC had a slightly higher proportion of males (59%), single (39%) or divorced/separated (10%) respondents, higher education and were living in public, 1–2 room housing (11%) as compared to the group without MHC. The mean number of CMCs in respondents with MHC (1.2) was lower than those without MHC (1.5).

**Table 1:** Profile of respondents without and with Mental Health Conditions (MHC)

Age	N	All		Without MHC		With MHC	
		**3077**		**2566**		**511**	
	Mean (SD)	45.7	0.32	47.1	0.35	38.6	0.69
**Age group (years)**	18–34	956	31%	720	28%	236	46%
	35–49	739	24%	604	24%	135	26%
	50–64	796	26%	697	27%	99	20%
	65 & above	586	19%	545	21%	41	8%
**Gender**	Male	1683	55%	1379	54%	304	59%
	Female	1394	45%	1187	46%	207	41%
**Ethnicity**	Chinese	817	27%	689	27%	128	25%
	Malay	981	32%	829	32%	152	30%
	Indian	1002	32%	824	32%	178	35%
	Others	277	9%	224	9%	53	10%
**Marital status**	Single	850	28%	652	25%	198	39%
	Married	1868	61%	1618	63%	250	49%
	Divorced/Separated	192	6%	140	6%	52	10%
	Widowed	167	5%	156	6%	11	2%
**Educational attainment**	Primary& below	519	17%	456	18%	63	12%
	Secondary/Junior College/ITE	1271	41%	1038	40%	233	46%
	Diploma/University	1287	42%	1072	42%	215	42%
**Employment**	Employed	2107	68%	1715	67%	392	77%
	Economically inactive	798	26%	723	28%	75	15%
	Unemployed	172	6%	128	5%	44	8%
**Housing Type**	Public, 1- 2 room	206	7%	152	6%	54	11%
	Public, 3 room	639	21%	540	22%	99	20%
	Public, 4 room	1046	35%	865	34%	193	36%
	Public, 5 room	803	27%	682	27%	121	24%
	Private, non-landed	225	7%	193	8%	32	7%
	Private, landed property	86	3%	74	3%	12	2%
**Having any CMC**		1786	58%	1508	59%	278	54%
**Number of CMC**	Mean (SD)	1.4	0.03	1.5	0.03	1.2	0.07

*Abbreviations*: *MHC* Mental Health Conditions, *ITE* Institute of Technical Education, *CMC* Chronic Medical Condition

Table 2 provides the average utilization and associated costs per year for respondents without and with MHC from 2017 to 2019. Since the respondents with MHC were notably different from those without MHC, with the former being relatively younger, we have provided the utilization and cost estimates stratified by different age groups. Table 2 also shows the age-adjusted estimates for those with MHC to increase comparability with those without MHC. There was a significant difference in the age distribution of respondents with and without MHC. The age-specific costs of respondents with MHC were generally higher, but since the age distribution was different across the two groups, the crude total costs were lower for the MHC group. Age-adjusted costs were derived by applying the age-specific costs of the ‘with MHC’ group to the age distribution of ‘without MHC’ group. Since age-adjusted costs are a better indicator of costs as the age distribution is held constant, these are provided in Table 2 along with crude costs. While respondents without MHC had an average total cost per year of S$1393.56, those with MHC had a 30% higher average total cost per year of S$1817.20. This difference in average total cost per year across two groups was highest for the inpatient admission costs (about 36% higher in MHC as compared to those without MHC), which was followed by ED (about 20% higher in MHC as compared to those without MHC) and SOC (about 18% higher in MHC as compared to those without MHC) related average cost per year. For primary care services, the average cost per year was 2% lower for those with MHC as compared to those without MHC.

**Table 2:** Average utilization and associated costs per year for respondents without and with MHC from 2017 to 2019

	**N**	**All**		**Without MHC**		**With MHC**		**With MHC**
		**3077**		**2566**		**511**		**Age adjusted **^**a**^
		**Mean**	**SE**	**Mean**	**SE**	**Mean**	**SE**	
**Total Cost**	All age groups	$1,369.10	81	$1,393.56	89	$1,246.26	201	$1,817.20
	18–34	$433.66	52	$424.64	55	$461.16	127	
	35–49	$720.36	116	$534.89	96	$1,550.15	461	
	50–64	$1,726.72	208	$1,778.71	225	$1,360.68	542	
	65 +	$3,227.52	253	$3,132.66	257	$4,488.48	1210	
**Inpatient Cost (Hospitalization)**	All age groups	$1,029.26	77	$1,044.62	84	$952.13	193	$1,424.37
	18–34	$284.02	49	$286.71	51	$275.81	125	
	35–49	$541.17	111	$371.38	90	$1,300.84	448	
	50–64	$1,320.32	200	$1,370.82	216	$964.81	519	
	65 +	$2,465.22	241	$2,374.86	245	$3,666.36	1141	
**ED Cost**	All age groups	$37.78	2	$36.89	2	$42.25	6	$44.12
	18–34	$27.65	3	$24.21	3	$38.17	7	
	35–49	$26.63	4	$21.84	4	$48.07	15	
	50–64	$38.48	5	$38.38	5	$39.20	14	
	65 +	$67.40	6	$68.41	7	$53.88	21	
**SOC Cost**	All age groups	$207.43	8	$212.81	9	$180.43	17	$251.80
	18–34	$87.24	6	$81.57	7	$104.52	13	
	35–49	$110.60	11	$102.76	12	$145.68	28	
	50–64	$245.48	17	$245.15	19	$247.81	45	
	65 +	$473.95	26	$466.79	27	$569.11	121	
**Primary Care Cost**	All age groups	$94.63	3	$99.24	3	$71.45	6	$96.91
	18–34	$34.74	2	$32.15	3	$42.67	5	
	35–49	$41.96	3	$38.92	4	$55.56	9	
	50–64	$122.44	7	$124.37	7	$108.85	16	
	65 +	$220.96	10	$222.60	10	$199.13	43	

*Abbreviations*: *MHC* Mental Health Condition, *ED* Emergency Department, *SOC* Specialist Outpatient care

^a^Adjusted to the age distribution in the ‘Without MHC’ group

Table 3 presents the multivariable analysis findings for the total healthcare expenditure. The unadjusted coefficient for MHC was not significantly associated with total healthcare expenditure in Model 1. However, on the addition of age group in Model 2, the association of MHC with total healthcare expenditure became significant (b = 0.376, *p* < 0.05), indicating the presence of MHC being associated with increased total healthcare expenditure after adjusting for age. The association of MHC with total healthcare expenditures was stronger (b = 0.508, *p* < 0.05) with the inclusion of other covariates in the final adjusted Model 3.

**Table 3:** Multivariable analysis findings for total healthcare expenditure

Total Cost per Year		**Model 1**		**Model 2**		**Model 3**		**Model 4**		**Model 5**	
		b	SE	b	SE	b	SE	b	SE	b	SE
**Any Mental Health Condition (MHC)**		-0.112	0.16	0.376*	0.174	0.508*	0.236				
**Age group** **(ref: 18–34)**	35–49			0.462**	0.171	0.065	0.195	1.117**	0.414	-0.233	0.203
	50–64			1.436***	0.168	0.809***	0.207	-0.0693	0.523	0.801***	0.212
	65 +			2.062***	0.184	0.885***	0.265	1.023	0.743	0.733**	0.266
**Sex (ref: Male)**	Female					0.00419	0.139	-0.0278	0.32	0.0218	0.141
**Race** **(ref: Chinese)**	Malay					0.0642	0.185	-0.0333	0.441	0.127	0.189
	Indian					0.425*	0.186	0.939*	0.421	0.35	0.190
	Others					0.0311	0.274	0.641	0.655	0.0669	0.281
**Education** **(ref: Primary and below)**	Secondary/ Junior College/ ITE					-0.0198	0.207	0.117	0.509	-0.0151	0.211
	Diploma/Degree					-0.554*	0.225	-0.214	0.558	-0.523*	0.231
**No. of CMC**						0.321***	0.052	0.303**	0.109	0.335***	0.050
**Interaction term**^a^	MHC*No. of CMC					-0.135	0.105				
	Constant	7.240***	0.0652	5.986***	0.118	5.917***	0.284	5.545***	0.685	5.970***	0.288
	Observations	3077		3077		3077		511		2566	

*Abbreviations*: *MHC* Mental Health Condition, *ITE* Institute of Technical Education, *CMC* Chronic Medical Condition

Models 1, 2 and 3: All respondents; Model 4: Respondents with MHC; Model 5: Respondents without MHC

^a^Interaction term between any MHC and Number of CMC

^*^p < 0.05, ** *p* < 0.01, *** *p* < 0.001

The coefficient of the interaction term between MHC and the number of CMC indicated that in respondents with MHC, the additional effect of having CMC on total healthcare expenditure was lower as compared to those without MHC (b = -0.135). However, this was not statistically significant. This interaction between co-occurring MHC and CMC is further explained by findings from Models 4 and 5, which were limited to respondents with and without MHC. In respondents with MHC, presence of CMC increased the total healthcare expenditure by 35% (exp(0.303) = 1.3539). Respondents without MHC had a 40% (exp(0.335) = 1.3979) increase in total healthcare expenditure with presence of CMC. In both Models 4 and 5, the association of the number of CMC with total healthcare expenditure was statistically significant.

While age was significantly associated with total healthcare expenditure across Models 3, 4 and 5, the pattern of association between age and total healthcare expenditure varied when the respondents were stratified into with MHC (i.e., Model 4) and without MHC (Model 5). In respondents without MHC, those in 50 to 64 years of age (b = 0.801, *p* < 0.001, exp(0.801) = 2.2278) or 65 years and above (b = 0.733, *p* < 0.01, exp(0.733) = 2.0813) had almost 2 times higher total healthcare expenditure as compared to those in the 18 to 34 years age group. This pattern of association was similar to the one observed in the total respondents' group or Model 3. Interestingly, in respondents with MHC, those in the relatively younger age group of 35 to 49 years (b = 1.117, *p* < 0.001) had almost 3 times higher total healthcare expenditure as compared to those in the 18 to 34 years age group.

Table 4 presents the multivariable analysis findings for different types of acute (i.e., inpatient and ED costs) healthcare expenditure, as a continuation of findings from Model 4 and 5 from Table 3. In respondents without MHC, those 50 to 64 years of age (b = 0.950, *p* < 0.02) or 65 years and above (b = 0.738, *p* < 0.05) had almost 2.6 and 2.1 times higher inpatient healthcare expenditure as compared to those in the 18 to 34 years age group. Interestingly, in respondents with MHC, those in the relatively younger age group of 35 to 49 years (b = 2.018, *p* < 0.05) had almost 7.5 times higher inpatient healthcare expenditure as compared to those in the 18 to 34 years age group. Table 5 presents the multivariable analysis findings for different types of outpatient (i.e., PC and SOC costs) healthcare expenditure. In respondents without MHC, those 50–64 years of age or 65 years and above had higher SOC and PC healthcare expenditure as compared to those in the 18–34 years age group. In respondents with MHC, while those in the age group of 35 to 49 years have lower SOC and PC healthcare expenditure than those in the 18 to 34 years age group, this association was not statistically significant.

**Table 4:** Multivariable analysis findings for different types of acute healthcare expenditure

Total Cost per Year		Inpatient Cost				Emergency Department Cost			
		Without MHC		With MHC		Without MHC		With MHC	
		b	SE	b	SE	b	SE	b	SE
**Age group** **(ref: 18–34)**	35–49	-0.308	0.283	2.018*	0.788	-0.635***	0.192	-0.653	0.364
	50–64	0.950**	0.297	-0.0735	1.001	-0.382	0.202	-1.221**	0.453
	65 +	0.738*	0.374	1.481	1.258	-0.184	0.235	-1.017	0.674
**Sex(ref: Male)**	Female	-0.0234	0.196	-0.287	0.544	0.0557	0.138	0.500	0.319
**Race** **(ref: Chinese)**	Malay	0.180	0.265	0.620	0.890	0.772***	0.177	0.696	0.382
	Indian	0.451	0.268	1.890*	0.866	0.886***	0.176	0.953*	0.370
	Others	0.223	0.395	1.699	1.253	0.585*	0.264	0.997	0.601
**Education** **(ref: Primary)**	Secondary/ Junior College / ITE	-0.0148	0.296	0.635	0.899	0.0767	0.196	-0.825	0.467
	Diploma/ Degree	-0.604	0.326	-0.0770	0.951	-0.781***	0.213	-1.759***	0.487
**No. of CMC**		0.354***	0.069	0.329	0.190	0.323***	0.045	0.281*	0.110
	Constant	5.551***	0.404	3.877**	1.321	2.692***	0.277	3.807***	0.562
	Observations	2566		511		2566		511	

*Abbreviations*: *MHC* Mental Health Condition, *ITE* Institute of Technical Education, *CMC* Chronic Medical Condition

^*^p < 0.05, ** *p* < 0.01, *** *p* < 0.001

**Table 5:** Multivariable analysis findings for different types of outpatient healthcare expenditure

**Total Cost per Year**		**Primary Care Cost**				**Specialist Outpatient Care Cost**			
		**Without MHC**		**With MHC**		**Without MHC**		**With MHC**	
		b	SE	b	SE	b	SE	b	SE
**Age group** **(ref: 18–34)**	35–49	-0.185	0.105	-0.247	0.197	-0.0333	0.123	-0.150	0.232
	50–64	0.456***	0.114	-0.076	0.243	0.604***	0.128	-0.156	0.281
	65 +	0.585***	0.142	0.147	0.366	0.838***	0.157	0.157	0.406
**Sex (ref: Male)**	Female	0.0596	0.074	0.131	0.157	0.123	0.0871	0.237	0.187
**Race** **(ref: Chinese)**	Malay	0.253*	0.100	0.407	0.208	-0.0453	0.115	-0.233	0.246
	Indian	0.224*	0.098	0.170	0.202	0.177	0.114	0.174	0.234
	Others	-0.519***	0.146	-0.636*	0.286	-0.208	0.169	0.167	0.333
**Education** **(ref: Primary)**	Secondary/ Junior College / ITE	-0.129	0.110	-0.337	0.256	0.0498	0.127	0.0143	0.293
	Diploma/ Degree	-0.463***	0.121	-0.625*	0.276	-0.141	0.137	-0.076	0.324
**No. of CMC**		0.392***	0.030	0.416***	0.067	0.280***	0.031	0.378***	0.069
	Constant	3.427***	0.149	3.768***	0.307	4.226***	0.170	4.403***	0.375
	Observations	2566		511		2566		511	

*Abbreviations*: *MHC* Mental Health Condition, *ITE* Institute of Technical Education, *CMC* Chronic Medical Condition

^*^p < 0.05, ** *p* < 0.01, *** *p* < 0.001

## Discussion

Our study found that the presence of MHC significantly increased the total healthcare expenditure of respondents by about 66% (exp(0.508) = 1.6620) as compared to those without MHC after adjusting for the covariates in an Asian setting (including adjusting for presence of CMC). Our findings are in agreement with existing literature that supports presence of MHC being associated with increased healthcare expenditure resulting in financial burden on the healthcare system. A Canadian based study reported significantly higher healthcare resource utilization and associated expenditure in respondents with MHC as compared to those without MHC [5]. Another observational study conducted in England reported the mean annual healthcare costs for people with serious mental illness to be £4989, with mean annual cost attributable to mental health as opposed to physical health being £2576 [4]. Wolff and colleagues examined the additional financial burden associated with psychiatric comorbidities in a hospital setting in Germany. Aligned with other similar studies, they reported psychiatric comorbidities to be associated with 40% additional hospital cost per episode [6].

In accordance with existing literature [4, 5], the presence of CMC was significantly associated with increased healthcare expenditure in both groups of respondents (those with versus without MHC). However, the magnitude of the additional healthcare expenditure due to CMC was higher in those without MHC (40%) as compared to those with MHC (35%). We would warrant caution at the optimistic interpretation of better management of co-occurring MHC and CMC, leading to lower costs in those with MHC. It may be possible that people with MHC and comorbid chronic physical conditions are not seeking the needed care [10]. Another consideration is that people with MHC may seek care from complimentary or alternative care settings, like Traditional Chinese Medicine, which is not captured in the administrative database. It is also reported that in low- to middle-income countries, care provided to people with MHC for comorbid chronic physical conditions may be inferior to the care provided to those without MHC [21]. We did not however find any evidence of any additive or synergistic effect of CMC on healthcare utilization.

Our results are in agreement with findings by Lee and colleagues, who reported that the incremental effect of CMCs on total healthcare expenditure is higher in patients without severe mental illness as compared to those with severe mental illness [9]. However, another recent study by Kaplan and colleagues involving 21,370 adults reported comorbid self-reported low mental health being associated with increased cost of treating a chronic medical condition [8]. This is contrary to our finding of the magnitude of increased expenditure associated with comorbid conditions being lower in respondents with MHC (exp(0.303) = 1.354) as compared to those without MHC (exp(0.335) = 1.398). Specifically, in participants with MHC, each additional CMC was associated with 35% increase in healthcare costs as compared to 40% increase in participants without MHC. Possible reasons could be related to the different definitions of mental health conditions (i.e., general mental health score versus specific diagnosed MHC), different composition of costs considered in both studies and different operationalization of comorbid conditions (i.e., each CMC considered separately versus as a group of CMC). Moreover, the authors concluded their reported estimates not to be substantial considering the smaller effect size [8].

Though past literature has consistently included age as a covariate in the analyses of healthcare utilization and costs related to MHC [4, 5, 19], none have described the varying association of age with total healthcare expenditure in those with and without MHC. To the best of our knowledge, we are the first to report differences in the pattern of association of age with total and specific types of healthcare expenditure in respondents with and without MHC. Specifically, respondents with MHC in 35–49 years age group had almost 3 times higher total healthcare expenditure and 7.5 times higher inpatient healthcare expenditure as compared to the 18–34 years age group. Our findings suggest an episodic, high cost, acute healthcare utilization pattern in the 35–49 year old respondents with MHC compared to 18–34 year old respondents after adjusting for comorbidities and socio-demographic covariates. This is somewhat unique to the group with MHC as the group without MHC follows the expected overall trend of higher age groups (50–64 and 65 and above) having higher overall and acute healthcare expenditure. One possible explanation of this finding could be related to the treatment gap associated with mental health conditions compared to other health disorders [22], which can result in delayed episodic utilization of more costly healthcare resources. Within Singapore, the treatment gap for severe mental conditions is reported to be about 78.9% [10]. This high prevalence of treatment gap in Singapore can be due to MHC related stigma. Moreover, it has been reported that stigma can drive the underutilization of healthcare services [23]. Within Singapore, researchers have illustrated that psychosocial attribution of MHC is associated with lower stigma and greater help seeking behaviour. However, younger people in the 18–34 years age group were more likely to demonstrate this psychosocial attribution of MHC than those in 35–49 years of age group [24]. The authors not only concluded stigma related delayed help seeking to be more prevalent in respondents in 35–49 years age group, but their findings also substantiate our hypothesis of potentially delayed, episodic, high cost, acute healthcare utilization in the 35–49 year old respondents with MHC.

While respondents with MHC in the 35–49 years age group had lower SOC and PC related healthcare expenditure as compared to those in the 18–34 years age group, this was not statistically significant. A recent study from Finland compared the PC utilization in those with depressive symptoms versus those without, using a matched control study design and reported those with MHC to have 3 times higher use of primary health care services as compared to those without depressive symptoms [19]. The difference in primary care utilization pattern across both studies could be due to relatively older participants (with a mean age of 51 years) in this study who may be either more open to seeking care for MHC (as compared to respondents in our study who had a mean age of 38.6 years) or may have more regular touchpoints with the healthcare system due to age-related healthcare needs. Another possibility could be related to reduced stigma and more willingness to seek care for MHC in a Western setting as compared to our Asian setting where MHC related stigma is highly prevalent [25].

There are several practical recommendations from our work. Considering the financial burden of significantly increased healthcare costs associated with MHC, timely planning and adequate resource allocation are needed to implement upstream measures for prevention, early diagnosis, and appropriate MHC management. Since comorbid chronic physical conditions were associated with increased healthcare expenditure in respondents with MHC, it would be beneficial to pro-actively screen people with MHC for chronic physical conditions to ensure early care and regular follow-up, which may potentially limit the costs incurred downstream. To increase help seeking behaviour of people with MHC, it is imperative to address the dual issues of treatment gap and high prevalent stigma. Adopting a tailored approach involving specific age groups of people with MHC, focusing on increasing awareness about psychosocial attribution of MHC and reducing stigma may be beneficial. Evidence-based contact interventions and educational interventions reported to effectively mitigate stigma [26] should be contextualized and adopted in the local setting. Recent efforts in this direction focussed on university students and single mental health condition (i.e., depression) have shown promising short-term results [27]. It is important to implement such interventions across different settings and determine long-term effectiveness. Reduction of the treatment gap for MHC will hopefully reduce episodic, high cost, acute healthcare utilization and associated expenditures.

Our study has several strengths. We are among the first few to not only describe the association of MHC with total healthcare expenditure in an Asian setting, but also report (a) the variations in association of CMC with total healthcare expenditure and (b) the variations in association of age with total healthcare expenditure in those with versus without MHC in an Asian setting. The linkage of administrative and survey data allowed us to exploit the relative strengths of both databases. The survey data provided information on respondent demographics, ethnicity, marital status, education and employment status, which allowed us to examine the association between these factors and the excess cost burden of respondents with MHC. The use of administrative data to track healthcare utilization allowed us to accurately monitor and track expenditures prospectively rather than rely on survey participants' recall, which may have introduced information bias. The ascertainment of the presence of CMC was based on two different data sources (i.e., survey and administrative), which increased the accuracy of data capture. We adopted a comprehensive approach to studying healthcare utilization by covering multiple healthcare services across both acute and outpatient settings. This comprehensive coverage also enabled us to provide the determinants of the overall healthcare cost and cost related to different types of healthcare services. Participants were interviewed in their preferred language in the SMHS 2016, which resulted in a more representative and inclusive sample.

There are several limitations of our study. Firstly, exclusion of institutionalized residents from the SMHS may have influenced the estimated prevalence of MHC as these groups are likely to have higher rates of mental disorders as compared to the general population. This limits the generalizability of survey findings to non-institutionalized residents. Secondly, with a response rate of 69.5% [28], about 30% of the approached population declined to participate, which may introduce a non-response bias. Another limitation is related to the unequal sample size in the MHC and without MHC group which may have potential implications on the difference in the mean number of CMCs across both these groups. However, the unequal sample size of the MHC and without MHC group did not impact the mean number of CMCs in both these groups based on a sensitivity analysis conducted on a random sample of 511 respondents from the group without MHC. The mean number of CMCs in those with MHC as compared with those without MHC did not change across the complete sample of 2566 respondents without MHC versus a random sample of 511 respondents without MHCs. The results of sensitivity analysis are provided in Supplementary Table 3 in the Additional File 1. As we captured the total healthcare utilization and associated expenditure of respondents with MHC, we did not specifically capture MHC related healthcare utilization and associated expenditure. The severity of CMC was not captured, which may influence the healthcare seeking by respondents. Hence, differences in severity of CMC in those with MHC versus those without could not be explored within the current manuscript. While using real-world data improves external validity, the internal validity may be influenced be above limitations and hence the interpretation of the findings should be done considering the influence of grouping variables.

## Conclusion

Our study reported that the presence of MHC significantly increased the total healthcare expenditure of respondents by about 66% as compared to those without MHC after adjusting for the covariates in an Asian setting. Additionally, we contributed new knowledge by reporting the variations in association of CMC with total healthcare expenditure and the variations in association of age with total healthcare expenditure in those with versus without MHC in an Asian setting. We reported that the magnitude of the additional healthcare expenditure due to CMC was higher in those without MHC (40%) as compared to those with MHC (35%) which may be explained by the prevalent high treatment gap and stigma in the local setting. Additionally, respondents with MHC in 35–49 years age group had almost 3 times higher total healthcare expenditure and 7.5 times higher inpatient healthcare expenditure as compared to the 18–34 years age group, suggesting an episodic, high cost, acute healthcare utilization pattern in the 35–49 year old respondents with MHC. The practical recommendations from our work are as follows: appropriate planning and resource allocation to implement upstream measures for prevention, early diagnosis and appropriate management of MHC, proactive screening of people with MHC for chronic physical conditions to ensure early care and regular follow-up and addressing dual issues of treatment gap and stigma to improve help seeking behaviour of people with MHC.

## Supplementary Information

**Additional file 1.**

**Additional file 2.**

## Data Availability

The datasets used and analysed during the current study are available from the last author on reasonable request. The datasets generated and/or analysed during the current study are not publicly available because the national claims data obtained from the Ministry of Health of Singapore (for the purpose of the current study) are not an openly available/accessible data source.

## Abbreviations

CHAS: Community Health Assist Scheme
CMC: Chronic Medical Condition
DALYs: Disability-adjusted life years
ED: Emergency department
GAD: Generalised anxiety disorder
GLM: Generalized Linear Models
GP: General practitioner
MHC: Mental health conditions
MOH: Ministry of Health
OCD: Obsessive compulsive disorder
PC: Primary care
SOC: Specialist outpatient services
SMHS: Singapore Mental Health Study
YLD: Years lived with disability
