# Supplementary material for: A prospective observational study exploring the association of comorbid chronic health conditions with total healthcare expenditure in people with mental health conditions in an Asian setting
Source: BMC Psychiatry. 2022 Mar 19;22:203. doi: 10.1186/s12888-022-03827-0 (PMC8933898; doi:10.1186/s12888-022-03827-0)
Supplement: Supplementary file 2 — Additional file 2. [file 12888_2022_3827_MOESM2_ESM.pdf]

Supplementary Table 1. Profile of Respondents without and with Mental Health Conditions (MHC) including those with Chronic Medical Condition (CHC) and those without CHC.

		**All**		**With MHC , n=511**				**Without MHC, n=2566**			
				**With CHC**		**Without CHC**		**With CHC**		**Without CHC**	
		**N=3077**		**278**		**233**		**1508**		**1058**	
		**N**	**%**	**N**	**%**	**N**	**%**	**N**	**%**	**N**	**%**
**Age**	mean (SD)	45.66	0.32	43.77	0.96	32.28	0.82	53.86	0.43	37.39	0.43
**Age group (years)**	18_34	956	31%	90	32%	146	63%	235	16%	485	46%
	35_49	739	24%	81	29%	54	23%	273	18%	331	31%
	50_64	586	26%	69	25%	30	13%	501	33%	196	19%
	>=65	2107	19%	38	14%	3	1%	499	33%	46	4%
**Gender**	Male	174	55%	174	63%	130	56%	845	56%	534	50%
	Female	104	45%	104	37%	103	44%	663	44%	524	50%
**Ethnicity**	Chinese	817	27%	62	22%	66	28%	372	25%	317	30%
	Malay	981	32%	79	28%	73	31%	507	34%	322	30%
	Indian	1002	33%	106	38%	72	31%	505	33%	319	30%
	Others	277	9%	31	11%	22	9%	124	8%	100	9%
**Employment**	Employed	2107	68%	212	76%	180	77%	923	61%	792	75%
	Economically inactive	798	26%	37	13%	38	16%	507	34%	216	20%
	Unemployed	172	6%	29	10%	15	6%	78	5%	50	5%
**Educational attainment**	Primary& below	519	17%	48	10%	15	6%	374	25%	82	8%
	Secondary/Junior College/ITE	1271	41%	131	17%	102	44%	652	43%	386	36%
	Diploma/University	1287	42%	99	47%	116	50%	482	32%	590	56%
**Housing Type**	Public, 1- 2 room	206	7%	39.00	14%	15.00	7%	100.00	7%	52.00	5%
	Public, 3 room	639	21%	67.00	25%	32.00	14%	324.00	22%	216.00	21%
	Public, 4 room	1046	35%	86.00	32%	95.00	42%	518.00	35%	347.00	34%
	Public, 5 room	803	27%	57.00	21%	64.00	28%	379.00	26%	303.00	29%
	Private, non-landed	225	7%	18.00	7%	14.00	6%	100.00	7%	93.00	9%

	Private, landed property	86	3%	6.00	2%	6.00	3%	56.00	4%	18.00	2%
**Marital Status**	Single	850	28%	72.00	26%	126.00	54%	243.00	16%	409.00	39%
	Married	1868	61%	159.00	57%	91.00	39%	1026.00	68%	592.00	56%
	Divorced/Separated	192	6%	37.00	13%	15.00	6%	102.00	7%	38.00	4%
	Widowed	167	5%	10.00	4%	1.00	0%	137.00	9%	19.00	2%
**No. Of CHC**	mean(SD)	1.44	0.03	2.17	0.10	0.00	0.00	2.53	0.04	0.00	0.00
Abbreviations: MHC: Mental Health Conditions; ITE: Institute of Technical Education; CMC: Chronic Medical Condition											

Supplementary Table 2. Average Utilization and Associated Costs per year for Respondents Without and With MHC including those with Chronic Medical Condition (CHC) and those without CHC from 2017 to 2019.

		**All**		**With MHC , N=511**						**Without MHC, N=2566**					
				**With CHC**			**Without CHC**			**With CHC**			**Without CHC**		
		**N=3077**		**N=278**			**N=233**			**N=1508**			**N=1058**		
		Mean	SE	Mean	SE	Age adjusted	Mean	SE	Age adjusted	Mean	SE	Age adjusted	Mean	SE	Age adjusted
**Total Cost**	All age groups	$1,369.10	81	$1,737.95	318	$1,419.92	$659.61	221	$1,189.66	$2,030.80	135	$1,026.98	$485.29	89	$344.69
	18-34	$433.66	52	$656.84	316		$340.54	62		$560.83	116		$358.65	60	
	35-49	$720.36	116	$1,422.08	464		$1,742.26	923		$930.74	203		$208.41	46	
	50-64	$1,726.72	208	$1,824.66	768		$293.53	182		$2,011.03	260		$1,184.88	440	
	65+	$3,227.52	253	$4,814.36	1292		$360.68	308		$3,344.74	277		$832.05	322	

**Inpatient cost (Hospitalization)**	All age groups	$1,029.26	77	$1,317.58	301	$1,065.10	$516.12	219	$1,068.21	$1,527.37	128	$752.87	$356.55	86	$230.85
	18-34	$284.02	49	$443.31	316		$172.55	53		$386.30	106		$238.46	56	
	35-49	$541.17	111	$1,065.63	431		$1,653.65	920		$681.55	192		$115.55	42	
	50-64	$1,320.32	200	$1,304.95	738		$182.51	183		$1,516.34	249		$998.85	429	
	65+	$2,465.22	241	$3,948.17	1221		$96.82	97		$2,538.57	265		$598.92	284	
**ED cost**	All age groups	$37.78	2	$54.79	10	$62.72	$27.28	5	$17.78	$51.35	4	$39.02	$16.27	2	$13.13
	18-34	$27.65	3	$41.08	12		$36.37	7		$32.33	5		$20.27	3	
	35-49	$26.63	4	$74.61	24		$8.27	4		$38.32	9		$8.24	2	
	50-64	$38.48	5	$48.89	19		$16.91	9		$46.53	7		$17.54	4	
	65+	$67.40	6	$55.73	23		$30.54	31		$72.28	7		$26.43	11	
**SOC cost**	All age groups	$207.43	8	$260.91	29	$208.52	$84.41	11	$74.81	$301.64	14	$162.24	$86.20	8	$77.75
	18-34	$87.24	6	$121.48	26		$94.06	15		$100.43	14		$72.44	7	
	35-49	$110.60	11	$200.82	43		$62.96	24		$147.74	23		$65.66	9	
	50-64	$245.48	17	$323.67	62		$73.33	25		$288.62	22		$134.01	34	
	65+	$473.95	26	$605.26	128		$111.11	111		$493.65	29		$175.36	48	
**Primary Care Cost**	All age groups	$94.63	3	$104.68	10	$83.58	$31.81	4	$28.85	$150.44	5	$72.86	$26.28	2	$22.95
	18-34	$34.74	2	$50.96	9		$37.56	5		$41.78	7		$27.48	3	
	35-49	$41.96	3	$81.02	14		$17.38	4		$63.13	7		$18.96	2	
	50-64	$122.44	7	$147.14	22		$20.78	8		$159.54	10		$34.48	5	

	65+	$220.96	10	$205.20	46		$122.21	70		$240.23	10		$31.33	7	
Abbreviations: MHC: Mental Health Condition; ED: Emergency Department; SOC: Specialist Outpatient care															

Supplementary Table 3. Mean Number of Chronic Medical Conditions (CMs) in those with MHC versus those without MHC.

**Sample**		**Without MHC**	**With MHC (N = 511)**	**P-value**
**Complete sample of those without MHC (N = 2566)**	Number of CMC, Mean (SD)	1.5	1.2	< 0.001
**Random sample of those without MHC (N=511)**	Number of CMC, Mean (SD)	1.4	1.2	0.015
Abbreviations: CMC: Chronic Medical Condition; MHC: Mental Health Condition				
